# Supplementary material for: Astrocyte deletion of Bmal1 alters daily locomotor activity and cognitive functions via GABA signalling
Source: Nat Commun. 2017 Feb 10;8:14336. doi: 10.1038/ncomms14336 (PMC5309809; doi:10.1038/ncomms14336)
Supplement: Supplementary Information — Supplementary Figures. [file ncomms14336-s1.pdf]

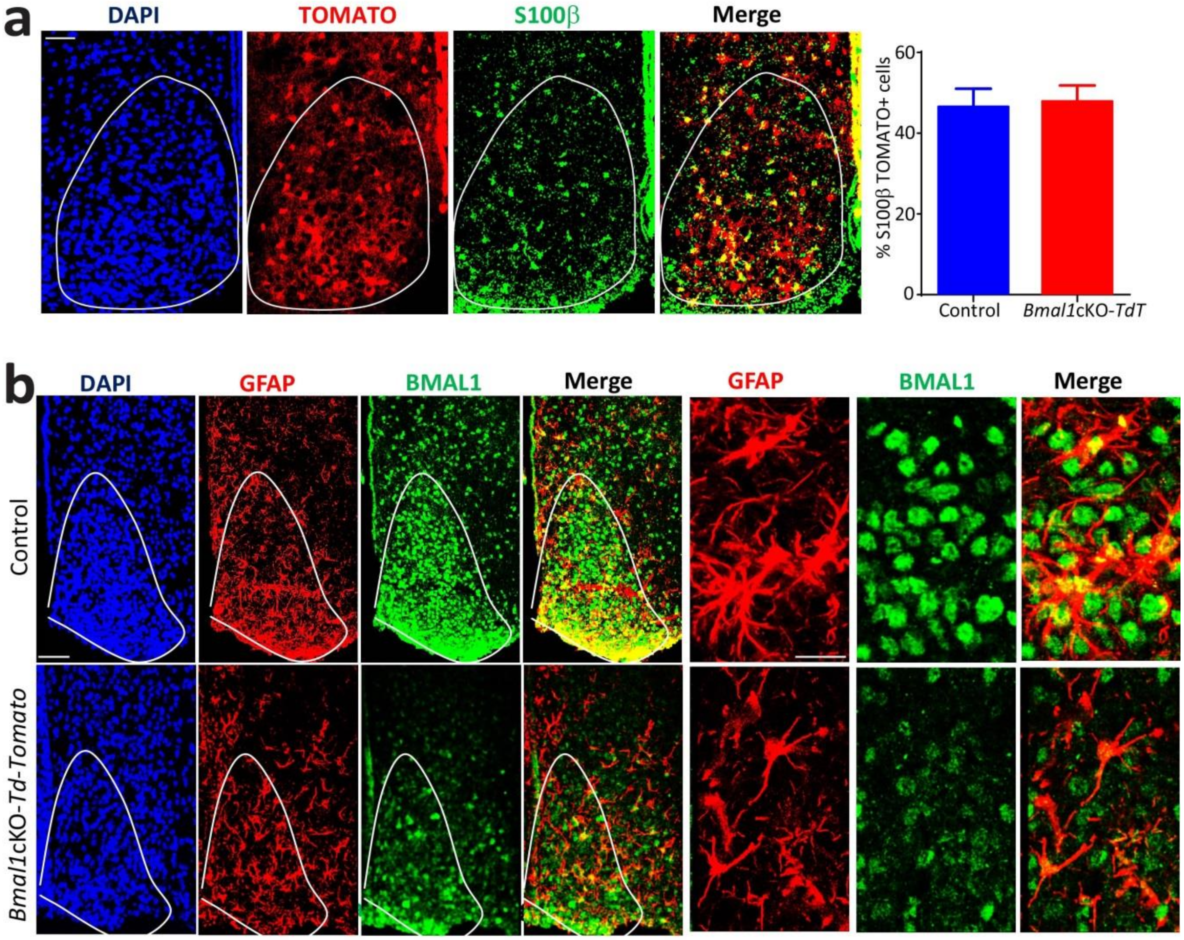

Supplementary Figure 1. Colocalization of Td-TOMATO cells and S100β**+ astrocytes and representative images of the colocalization of GFAP and BMAL1+ cells. (a)** *Glast:*CreER^{T2} mice drive the expression of Td-TOMATO in S100β positive astrocytes of the SCN. Representative micrographs of S100β immunostaining in the SCN (white line) of control mice (DAPI in blue, TOMATO in red and S100β in green). Quantification of the percentage of Td-TOMATO positive cells that co-localized with S100β is shown in the right panel. The value express the mean $\underline{+}$ s.e.m. (n=4 animals per group) (**b**) Representative micrographs of BMAL1 immunostaining in the SCN of control or *Bmal1*cKO-*Td-Tomato* animals in 12h:12h LD cycles (DAPI in blue, GFAP in red and BMAL1 in green). Scale bar: 50μm and 20μm in the higher magnification images.

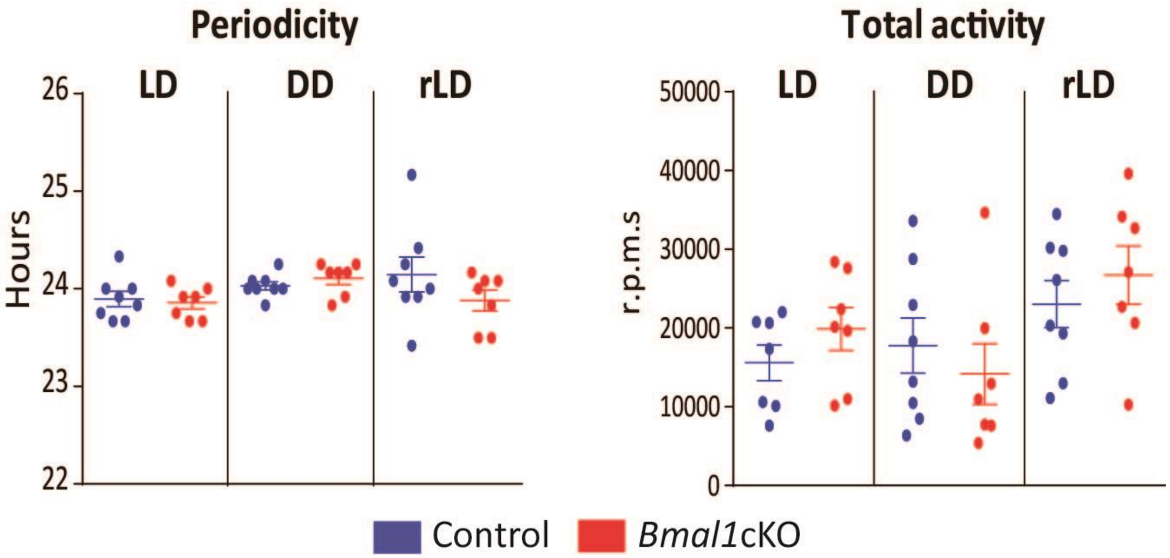

Supplementary Figure 2. Circadian period and total activity in *Bmal1*cKO and control animals. Circadian periodicity (left panel) and total activity (right panel) were largely retained in *Bmal1*cKO mice as compared with control animals in all lighting conditions with no significant differences between genotypes (paired t-test). The value express the mean $\pm$ s.e.m. (n=8 animals control and n=7 *Bmal1*cKO mice).

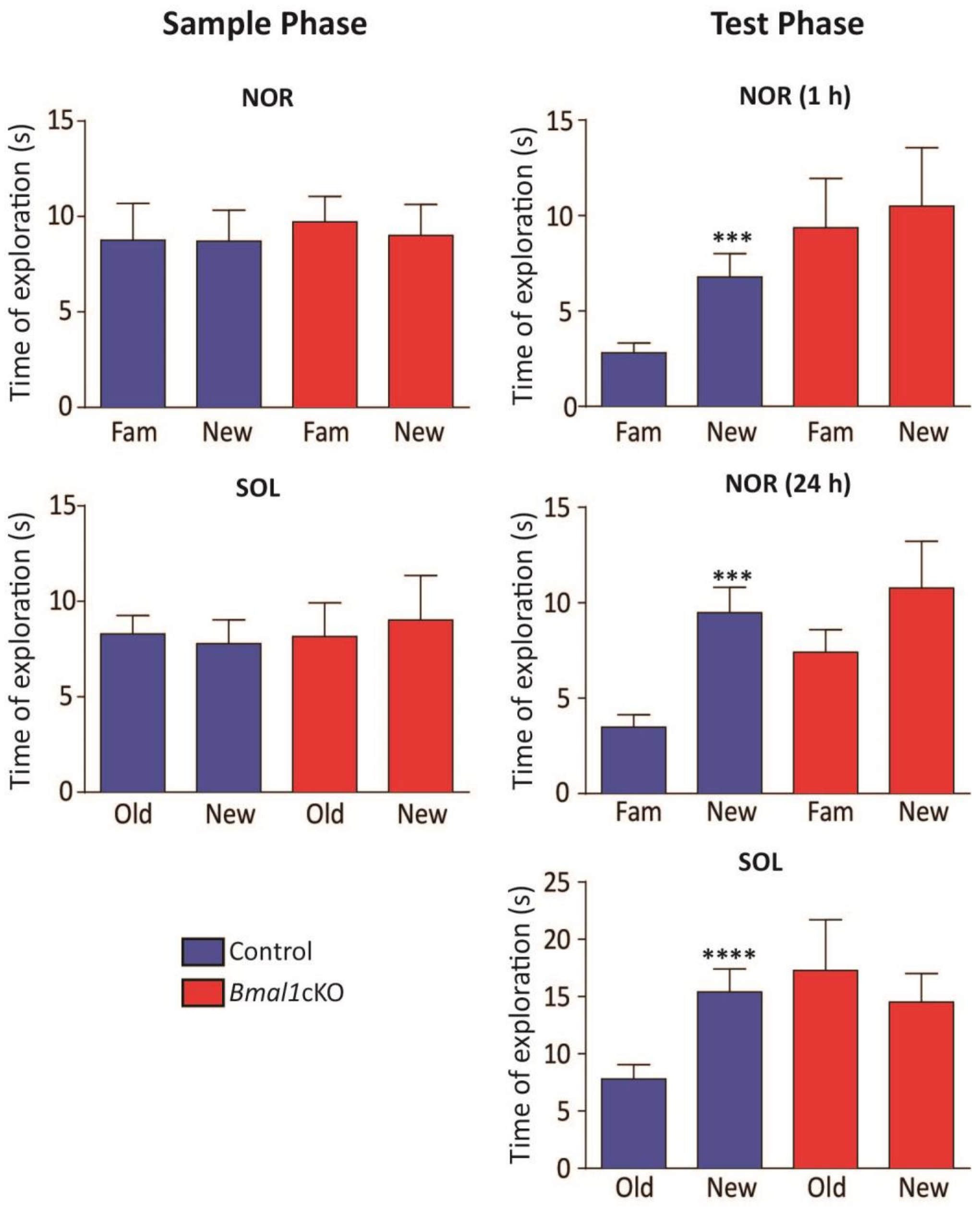

Supplementary Figure 3. Time of exploration of control and *Bmal1*cKO mice in the sample and test phases of the NOR and NOL tasks. Time of exploration in sample (left panel) and test (right panel) phase of control and *Bmal1*cKO in the NOR (1h and 24 h) and SOL**.** Both groups explored the familiar (Fam) and novel (New) object during familiarization of the NOR and SOL paradigms. Statistical analysis revealed a significant effect of object in the test phase of NOR (1h and 24 h) and SOL in control animals (paired t-test ***=p<0.001 and ****=p<0.0001 vs Fam object). However, *Bmal1*cKO did not differ significantly in exploration of both objects in the tasks. The values express the means ± s.e.m. (n=10 animals control and n=10 *Bmal1*cKO mice).

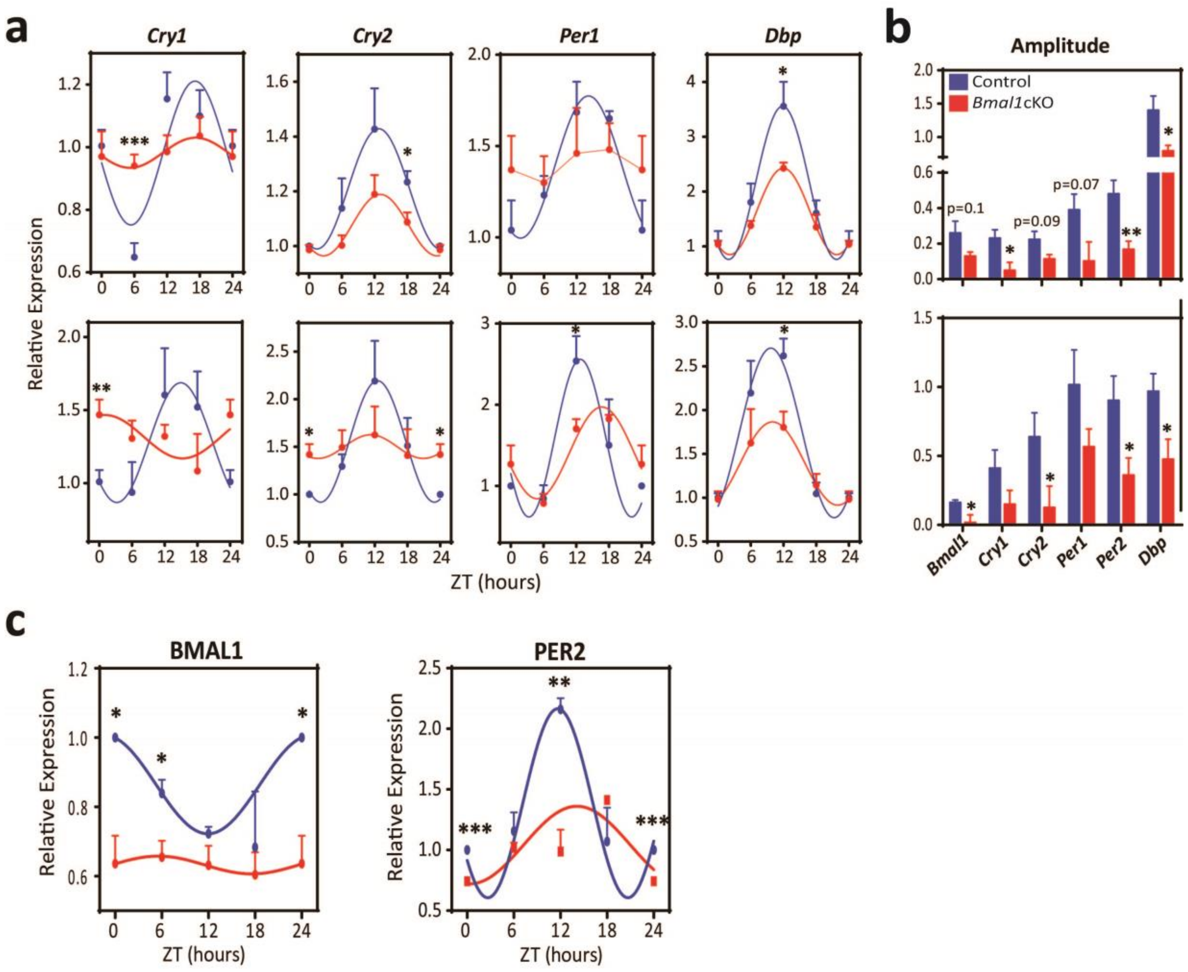

Supplementary Figure 4. Expression of circadian oscillators in cortex and hippocampus of control and *Bmal1*cKO by qPCR and western blot. (a) Oscillation of *Cyr1*, *Cry2*, *Per1* and *Dbp* by qPCR in cortex (upper panel) and hippocampus (lower panel) of control and *Bmal1*cKO mice. Samples were collected from mice under 12h:12h LD cycles. Note that the ZT24 time-point is the ZT 0 time point shown again. Graphs shown the mean ± s.e.m. of 5 animals per group at each time point (paired t-test, *p<0.05; **p<0,01 vs control animals). **(b)** Reduced amplitude of *Bmal1*, *Cry1*, *Cry2*, *Per1*, *Per2* and *Dbp* in cortex (upper panels) and hippocampus (lower panels) of *Bmal1*cKO mice, as compared to controls animals (paired t-test, *p<0.05; **p<0,01; ***p<0,001 vs control animals). **(c)** Quantification of cortical BMAL1 and PER2 western blots showing reduced levels of BMAL1 in *Bmal1*cKO mice as well as reduced oscillation in mutants as compared to control animals. The value express the mean ± s.e.m. of 3 animals per group and time point (paired t-test, *p<0.05; **p<0,01; ***p<0,001 vs controls).

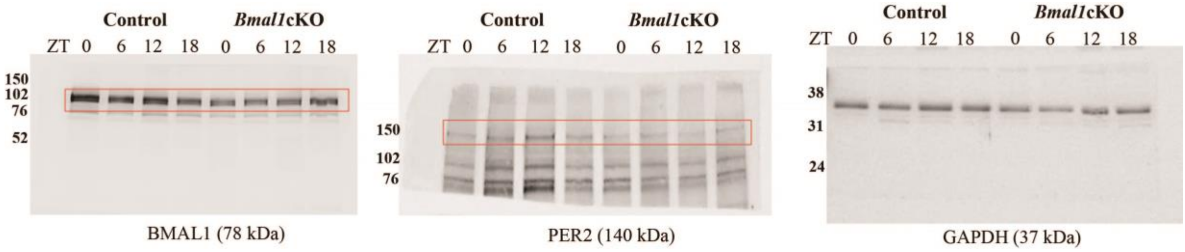

Supplementary Figure 5. Full western blots from which the cropped images in Figure 3c of the main paper were taken. The red boxes indicate the cropped areas.

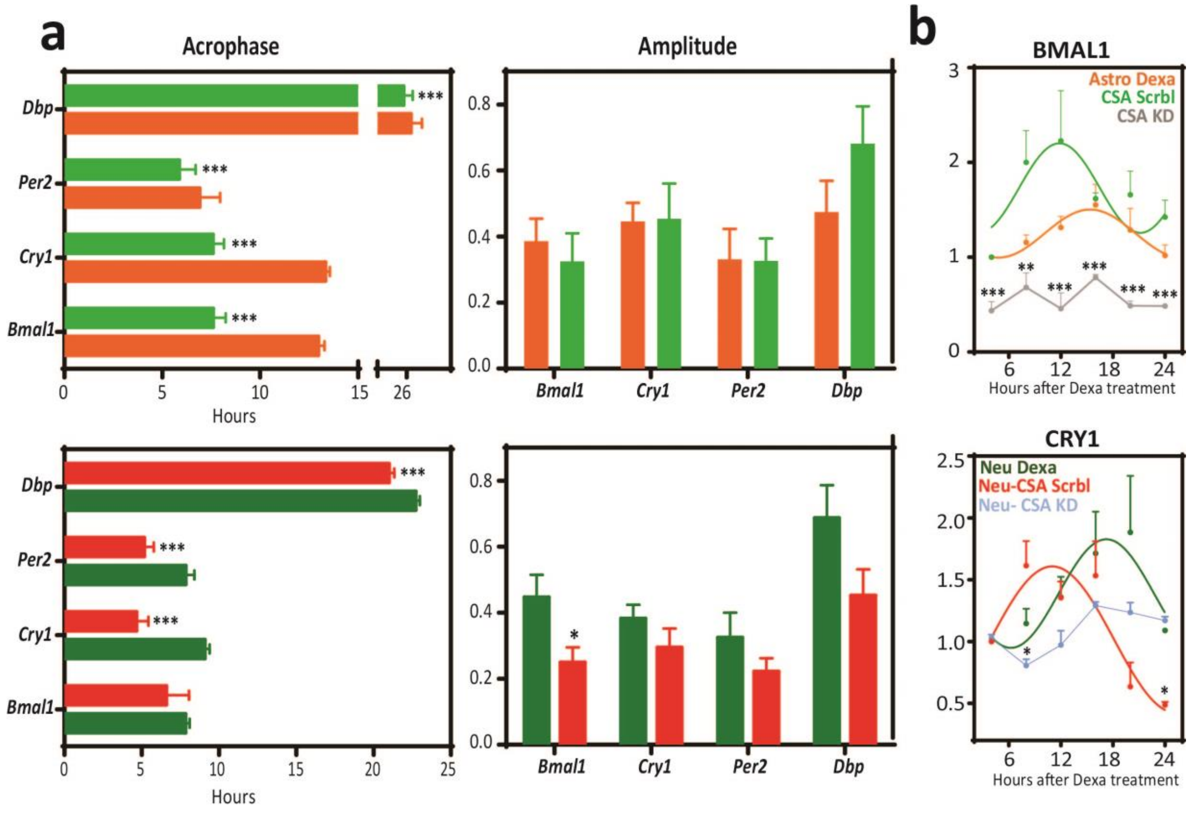

Supplementary Figure 6. Acrophase, amplitude and western blot quantification of circadian oscillators in astrocytes and neurons. (a) Acrophase (left panel) and amplitude (right panel) of *Bmal1*, *Cry1*, *Cry2*, *Per1*, *Per2* and *Dbp* in astrocytes (upper panel) and neurons (lower panel) synchronized with Dexamethasone or in co-culture. The value express the mean ± s.e.m. of three independent experiment performed in triplicate (paired t-test *p<0.05; **p<0,01; ***p<0,001 vs astrocytes or neurons treated with Dexamethasone). **(b)** Quantification of BMAL1 in primary astrocytes (up) or CRY1 in neurons (bottom), showing expression of BMAL1 in Dexamethasone-treated astrocytes in isolated cultures or Dexamethasone-treated astrocytes in co-culture with asynchronous neurons, upon transfection with scramble siRNAs (CSA Scrbl) or with Bmal1 siRNAs (CSA KD) (upper panel); (bottom panel) entrainment of CRY1 in cortical neurons after co-culture with Scrbl transfected synchronous astrocytes (Neu-CSA Scrbl) is not observed when co-culture is performed with arrhythmic astrocytes (Neu-CSA KD). The value express the means ± s.e.m. of two independent experiment performed in triplicate (paired t-test, *p<0.05; **p<0,01; ***p<0,001 vs CSA Scrbl or Neu-CSA Scrbl).

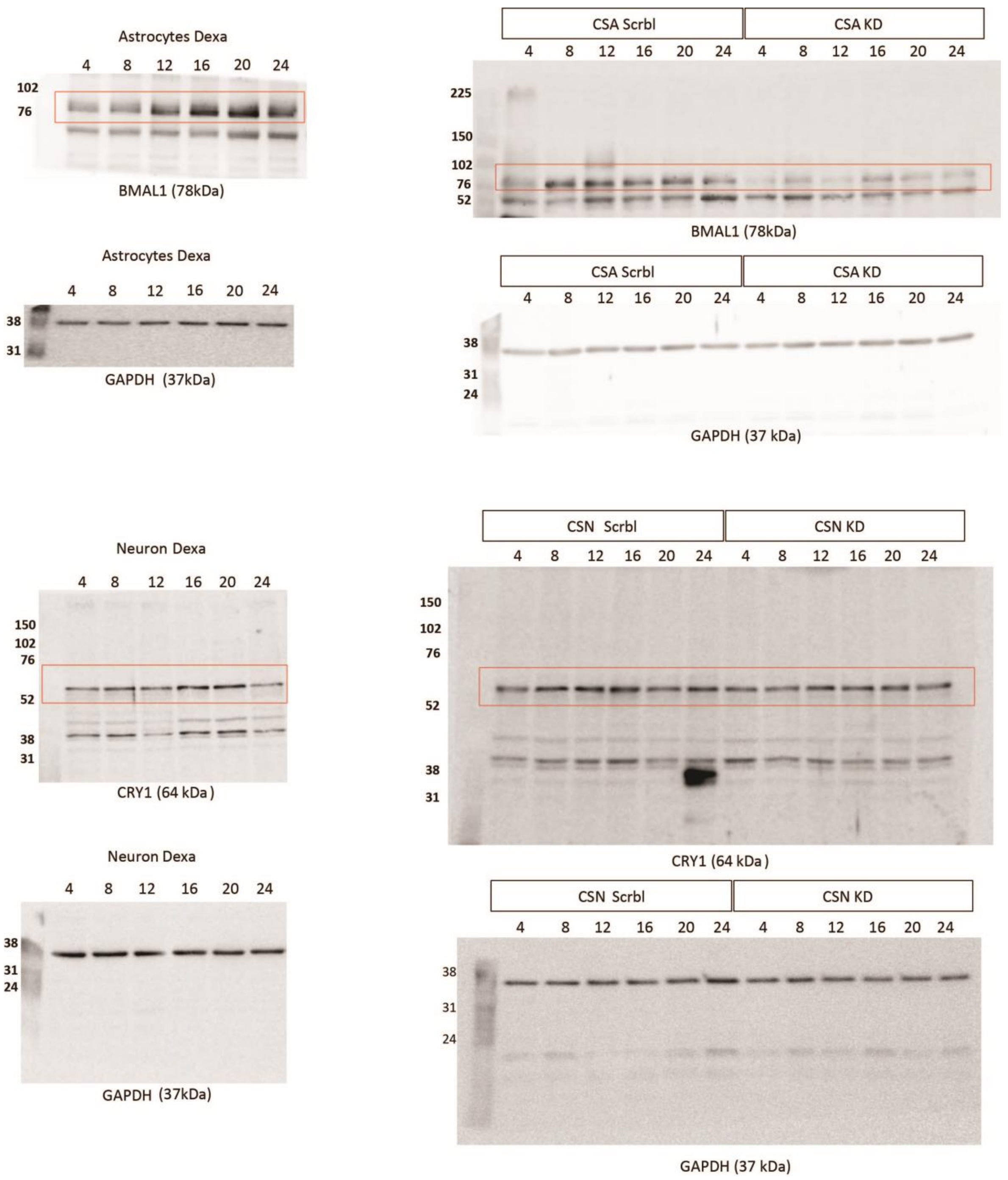

Supplementary Figure 7. Full western blots from which the cropped images in Figure 4c of the main paper were taken. The red boxes indicate the cropped areas.

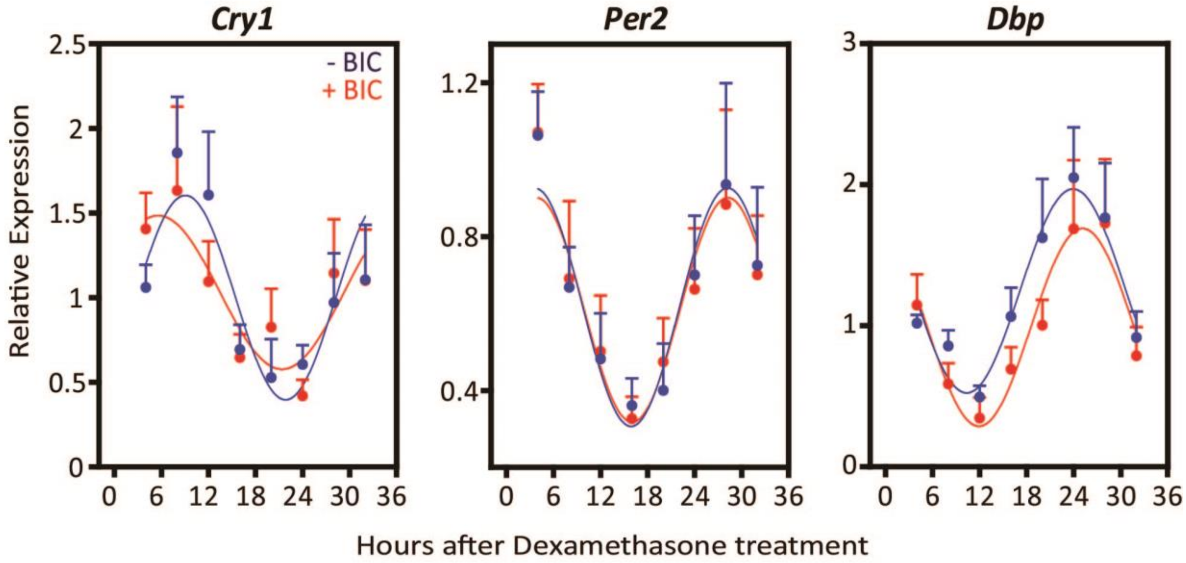

Supplementary Figure 8. Expression of *Cry1*, *Per2* and *Dbp* in astrocytes in coculture with neurons, in presence of Bicuculline. Primary cortical astrocytes were synchronized by Dexamethasone (100 nM) for 2 hours. After washing, astrocytes were placed in co-culture with asynchronous cortical neurons in presence of Bicuculline (30 μM) or vehicle. No statistical significant changes in the rhythmic oscillations of *Cry1*, *Per2* and *Dbp* by qPCR were observed in presence of Bicuculline. Graphs shows the means ± s.e.m. of three experiments performed in triplicate.

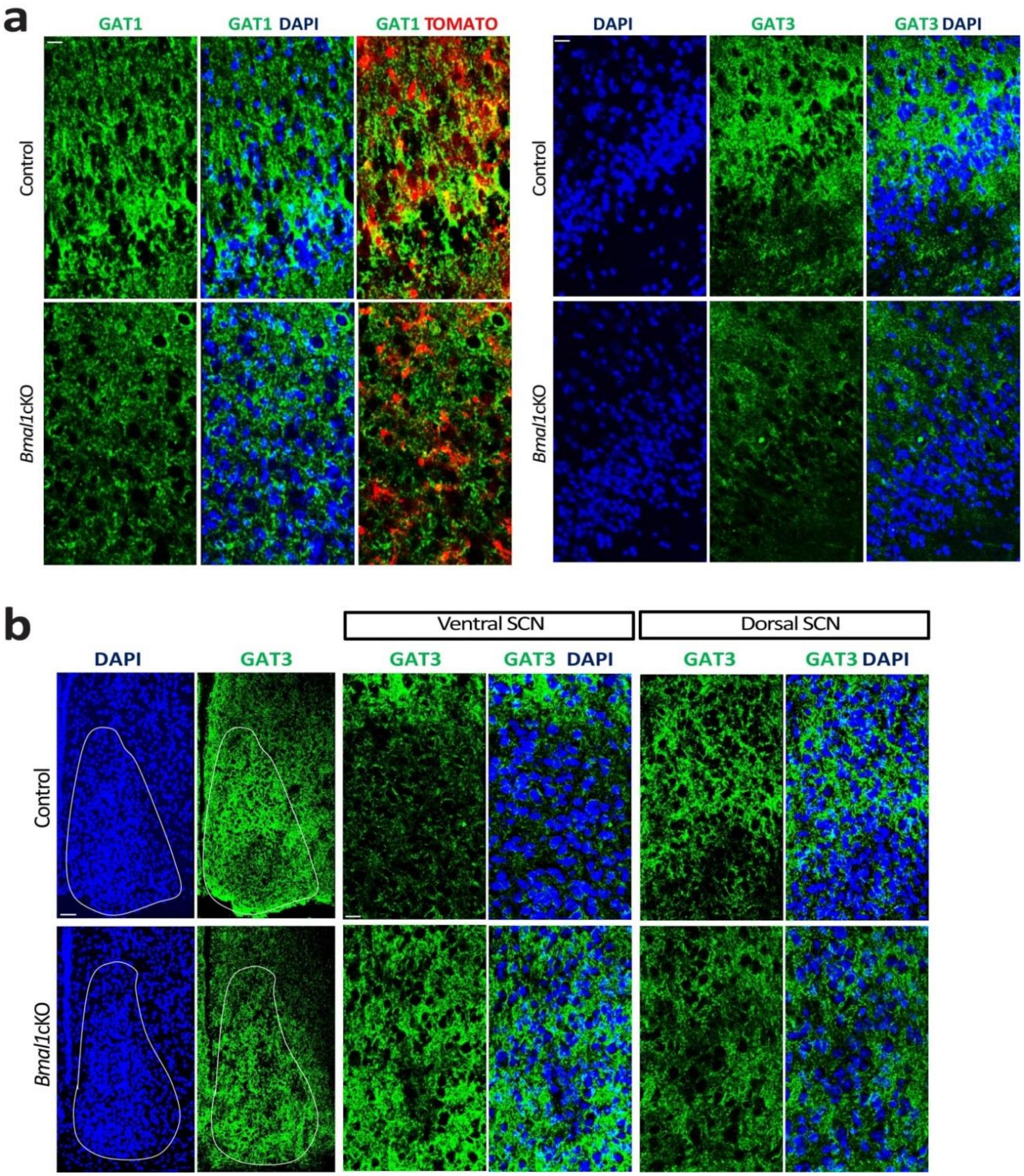

Supplementary Figure 9. Representative images of GAT1 and GAT3 immunostaining in cortex and SCN of control or *Bmal1*cKO mice. (**a**) Representative micrographs of GAT1 (left panel) and GAT3 immunostaining (right panel) in cortex of *Bmal1*cKO-*Td-Tomato* or *Bmal1*cKO, respectively, and control mice in 12h:12h LD cycles at ZT0. Scale bar: 20µm. **(b)** Representative micrographs of GAT3 immunostaining in the SCN of *Bmal1*cKO and control mice in 12h:12h LD cycles at ZT0. Scale bar: 50µm and 20µm in the higher magnification images. n=4 animals per group.

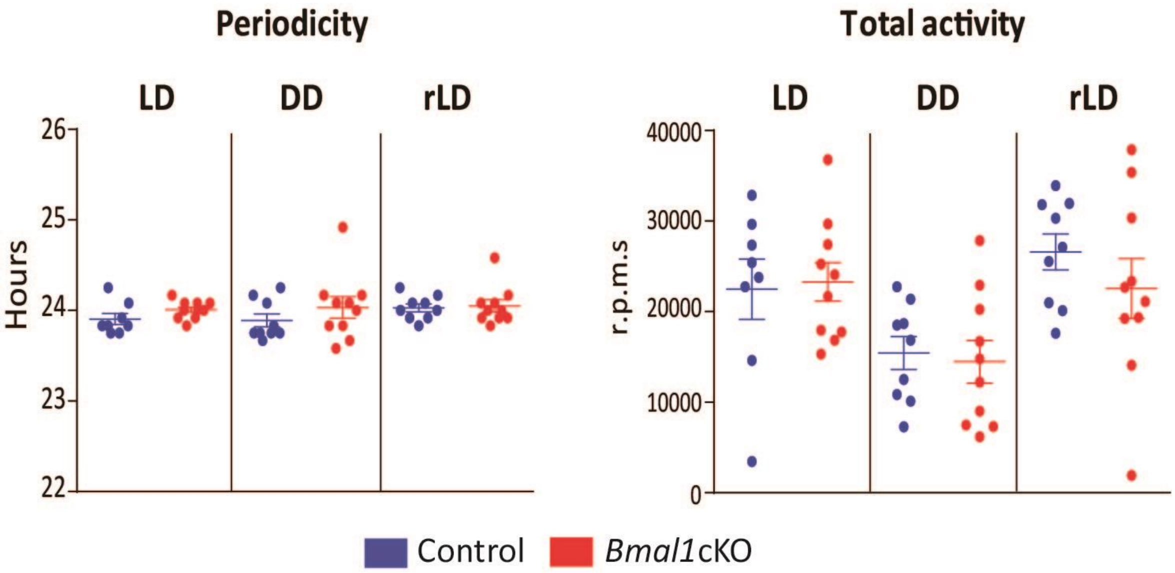

Supplementary Figure 10 . Periodicity and total activity of control and *Bmal1*cKO mice treated with PTZ. Circadian period (left panel) and total activity (right panel) in control and *Bmal1*cKO mice in all lighting conditions after PTZ administration, showing no significant differences between genotypes (paired t-test). The value express the mean ± s.e.m. (n=9 animals control and n=10 *Bmal1*cKO mice).

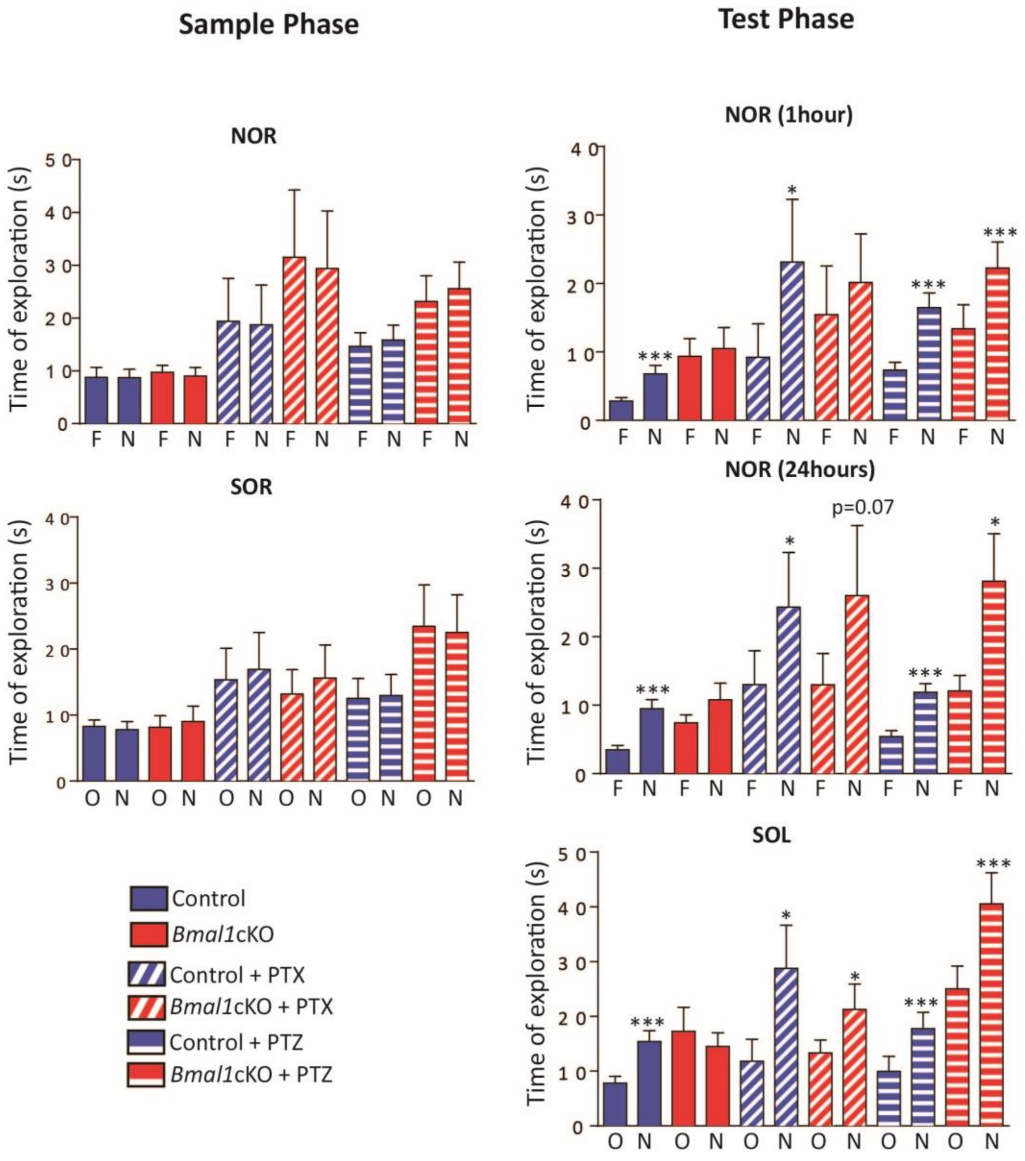

Supplementary Figure 11. Time of exploration of control and *Bmal1*cKO mice after PTZ or PTX treatments in the sample and test phases of the NOR and NOL tasks. Time of exploration in sample (left panel) and test (right panel) phase of control and *Bmal1*cKO mice in the NOR (1 h and 24 h) and SOL tasks, after PTZ or PTX administration. Both groups explored the familiar (F) and novel (N) object during familiarization of the NOR as well as the old (O) and new (N) object locations in the SOL paradigms. Statistical analysis revealed a significant effect of object in the test phase of NOR (1h and 24 h) and SOL in both control and *Bmal1*cKO animals (paired t-test, *=p<0.05; ***=p<0.01 vs Fam/Old). The value express the means ± s.e.m. (n=10 animals per group).
